# Interaction between c-jun and Androgen Receptor Determines the Outcome of Taxane Therapy in Castration Resistant Prostate Cancer

**DOI:** 10.1371/journal.pone.0079573

**Published:** 2013-11-08

**Authors:** Martina Tinzl, Binshen Chen, Shao-Yong Chen, Julius Semenas, Per-Anders Abrahamsson, Nishtman Dizeyi

**Affiliations:** 1 Department of Clinical Sciences, Lund University, Malmö, Sweden; 2 Department of Hematology-Oncology, BIDMC, Harvard Medical School, Boston, Massachusetts, United States of America; 3 Department of Laboratory Medicine, Lund University, Malmö, Sweden; AMS Biotechnology, United Kingdom

## Abstract

Taxane based chemotherapy is the standard of care treatment in castration resistant prostate cancer (CRPC). There is convincing evidence that taxane therapy affects androgen receptor (AR) but the exact mechanisms have to be further elucidated. Our studies identified c-jun as a crucial key player which interacts with AR and thus determines the outcome of the taxane therapy given. Docetaxel (Doc) and paclitaxel (Pac) agents showed different effects on LNCaP and LNb4 evidenced by alteration in the protein and mRNA levels of c-jun, AR and PSA. Docetaxel-induced phophorylation of c-jun occurred before JNK phosphorylation which suggests that c-jun phosphorylation is independent of JNK pathways in prostate cancer cells. A xenograft study showed that mice treated with Pac and bicalutamide showed worse outcome supporting our hypothesis that upregulation of c-jun might act as a potent antiapoptotic factor. We observed in our in vitro studies an inverse regulation of PSA- and AR-mRNA levels in Doc treated LNb4 cells. This was also seen for kallikrein 2 (KLK 2) which followed the same pattern. Given the fact that response to taxane therapy is measured by PSA decrease we have to consider that this might not reflect the true activity of AR in CRPC patients.

## Introduction

The treatment options for patients with castration resistant prostate cancer (CRPC) are still limited. Although new promising drugs like CYP17A1 inhibitor arbiraterone and MDV3100 have entered the market, taxane based chemotherapy is still considered worldwide the most important cornerstone of treatment when androgen deprivation therapy (ADT) has failed [1-4]. Besides the classic taxanes, Docetaxel and Paclitaxel, Cabazitaxel is used as chemotherapeutic agent in the treatment of CRPC patients, the latter mostly to treat patients with Doc resistant disease [5].

Taxanes arrest cells in the G2-M phase by hyperstabilization of the microtubules prompting the cells to cell death [6]. Besides this well described effect in tumor cells there is convincing evidence that taxane therapy also interferes with androgen receptor (AR) in prostate cancer cells [7-11]. Our group has identified c-jun as an important key player in this interaction between AR and taxanes which affects the outcome of treatment in the castration resistant status of prostate cancer cells.

Transcription factor c-jun is a proto-oncogene and belongs to the AP-1 family which consists of the jun, fos and ATF-2 subfamilies [12,13]. AP-1 proteins homo- or heterodimerize before binding to their DNA target site. The AP-1 proteins are multifunctional and involved in regulating stress response signals, cell growth and apoptosis [12]. Nevertheless, there are data which strongly suggest that c-jun is a growth promoter and proto-oncogene [14,15]. Shemshedini and coworkers showed that, similar to what observed with other AR coactivators, c-jun can mediate AR N-to-C interaction to enhance DNA binding [16,17]. Furthermore, phosphorylated c-jun is frequently overexpressed in human cancers [18,19] and has been linked to invasive properties of prostate and breast cancer [18,20,21]. However, the role of c-jun activation and possible interaction with AR in the cell fate after exposure to Doc is part of a complex network and remains to be elucidated. This study was carried out to investigate the consequence of interaction of c-jun and AR in taxane treatment of castration resistant prostate cancer cells. To optimize the efficacy of chemotherapy with taxanes we need to identify a specific molecule or pathway which may confer response or resistance. In the present study we sought to examine the effect of taxanes as single agent or in combination with bicalutamide on AR and its cofactor c-jun *in vitro* and *in vivo*. 

## Materials and Methods  

### Cell lines and Reagents

The human prostate cancer cell lines LNCaP and PC-3 were obtained  from the American Type Culture Collection (ATCC, Manassass, VA). LNb4 cells were generated in our laboratory from LNCaP cells exposed to bicalutamide (5 µM in serum reduced to 5%). Cells were cultured in RPMI (LNCaP) and Hams-F12 (PC-3) supplemented with 10% fetal bovine serum (FBS) and 1% penicillin-streptomycin (Life Technologies, Paisley, UK). Cells were treated with Docetaxel (Taxotere®, Sanofi Aventis) and Paclitaxel (Paclitaxel®, Actavis, Hafnarfjordeur, Iceland) in the concentration stated in each figure. Dihydrotestosterone (DHT) was purchased from Steraloids Inc. (Newport, RI) and bicalutamide from AstraZeneca (London, UK). All Western blot reagents were purchased from Invitrogen (Carlsbad, CA, USA) except JNK inhibitor XIV SR 3306 (Calbiochem, Darmstadt, Germany).

### Transfection and small interfering RNA (siRNA)

Transient transfections with c-jun, c-jun mutant (c-jun Ala63/73) and AR plasmids (all kindly provided by Shao-Yong Chen, Harvard Medical School) in PC-3 prostate cancer cells were performed using FuGENE 6 (Roche Diagnostics, Mannheim, Germany) as recommended by the manufacturer. The c-jun mutant (c-jun Ala63/73), herein referred as junA, used in experiments carries a mutation at the serine site 63/73 (Ala63/73) which is the major target of phophorylation by stress stimuli. For siRNA experiments, PC-3 and LNCaP cells were transfected for 48 hours with 100 nM siRNA c-jun or non-targeting siRNA (Cell Signaling Technology, Danvers, MA, USA). Transfection was performed using X-tremeGENE siRNA transfection reagent (Roche Diagnostics) according to manufacturers instruction.

### Proliferation

Cell viability was defined by trypan blue exclusion (Sigma-Aldrich, St Louis, MO, USA). PC-3, LNCaP and LNb4 cells were seeded in 24-well plates at a density of 25,000 cells per well. After 24 and 48 hours treatment with Doc or DMSO (Sigma-Aldrich) at the concentrations indicated floating and attached cells were collected by trypsinization. Equal portions of cell suspensions and 0.4% trypan blue were mixed, and the number of viable, trypan blue-excluding cells were counted by using a hemacytometer. The percentage of viable cells was expressed per 100 of the total cells (mean ± SD). transfected PC-3 cells were further evaluated by MTS assay. After 48 hours of transfection cells were exposed to Doc or remained untreated for further 48 hours. After incubation at 37 °C in 5% CO_2_ for 4 hours, the number of living cells was measured using an 3-(4,5-dimethylthiazol-2-yl)-5-(3-carboxymethoxyphenyl)-2-(4-sulfophenyl)-2H-tetrazolium (MTS) assay (Celltiter 96 Aqueous One-solution Cell Proliferation assay, Promega, Madison, WI, USA) according to the manufacturers instructions. Absorbance was measured at 490 nm using a multiwell plate reader (Model 680, Bio-Rad, Richmond, CA, USA), with wells containing only medium serving as blank controls. 

### Flow cytometry for cell cycle and apoptosis analyses

Cell cycle distribution was analyzed by flow cytometry. Briefly, transfected cells were treated with Doc for 48 hours and were fixed and stained with propidium iodide for 40 min. The viable cells were gated, and DNA content of at least 10 000 labeled cells was quantified with a fluorescence-activated cell sorter. Apoptosis was determined by Annexin V conjugated with fluorescein isothiocyanate (FITC) and 7-AAD staining (BD Pharmingen of BD Bioscience, San Jose, CA, USA). Cells were treated with Doc for 48 and 72 hours and stained cells were subjected to flow cytometric analysis on a FACSCalibur  (BD Bioscience, San Jose, CA, USA) instrument. Data presented are obtained from two independent experiments in duplicates and were analyzed using FCS Express software (DeNovo Software, Los Angeles, CA, USA). 

### Western Blot analysis and Immunoprecipitation

For Western blot analysis total protein was extracted using radioimmunoprecipitation assay (RIPA) buffer (50 mmol/L Tris-HCl, 150 mM NaCl, 20 mM EDTA, 1% NP40, 0.1%  SDS, 1 mM Na3VO4, 1 mM NaF and 1 mM phenylmethylsulfonylfluoride ) supplemented with the protease inhibitor cocktail Complete Mini ( Roche, Mannheim, Germany). Total protein concentration was measured using BCA protein assay (Pierce, Rockford, IL). Protein samples (20-30 µg) were loaded on 4-12 % SDS-PAGE and transferred to a nitrocellulose membrane Bio-Rad assay (Bio-Rad, Hercules, CA, USA) and subjected to electrophoretic analysis and blotting. Membranes were probed overnight at 4 °C with the following primary antibodies: anti-AR (441 and N-20), anti-c-jun, anti-p-cjun (ser 63/73), and  anti-β-actin, all purchased from Santa Cruz ( Santa Cruz Biotechnology,CA), anti-PSA (Dako, Glostrup, Denmark ), p-JNK from Cell Signaling Technology (Beverly, MA). Secondary antibodies (IRDye 680, IRdye 800) were obtained from Li-Cor Biotechnology (Nebraska, USA ) and proteins detected by Odyssey® Infrared Imaging System.

For immunoprecipitation cells were treated as indicated and harvested in lysis buffer. Cell lysates were homogenized, protein content measured and 500 μg of precleared protein was incubated with anti-AR antibody at 4°C overnight with continuous agitation. Equal amounts of mouse IgG was used as negative control. Protein G beads (Pierce, Rockford, IL, USA) were then added to each sample and incubated at 4 °C for 2 hours. Samples were then centrifuged at 2000 x g 2 min and Laemmli sample buffer (30 μl) without β-mercaptoethanol added and the mixture incubated at 65°C for 15 minutes to elute bound proteins. Eluate fractions were centrifuged at 2000 x g, 1μl of β-mercaptoethanol was added and samples subjected to Western blotting as described above. Protein expression was evaluated relative to β-actin expression by Densitometric analysis.

### Real-time Quantitative PCR Analysis  

Total RNA was isolated from the LNCaP and LNb4 cells using RNeasy Mini Kit (Qiagen, West Sussex, UK) following the manufacturers protocol and treated with RNase-free DNase (DNase I; Amersham Pharmacia Biotech, Sollentuna, Sweden) to remove potential genomic DNA contaminants. Each cDNA was synthesized by reverse transcription from 1 μg of total RNA using the StrataScript First-Strand Synthesis System and random hexamer primers (Stratagene; AH diagnostics, Stockholm, Sweden). Real-time PCR was performed with SYBR Green QPCR master mix ( iScript from BioRad)  in a MX 3000P detection system (Stratagene) with an initiation step at 95°C for 10 minutes followed by 40 cycles at 95°C for 30 seconds, 56°C for 1 minute and 72°C for 1 minute. 

The following primers for each gene were used: PSA (F5'-AGGCCTTCCCTGTACACCAA-3' and R5'-GTCTTGGCCTGGTCATTTCC-3'), AR (F 5'-GTACCTGTCAGCCCCTGAAC-3' and R5' GGAGAGCTGCT TTCG- CTTAG-3'), GAPDH (5' -CGA CCA CTT TGT CAA GCT CA-3' and 5'-AGG GGT CTA CAT GGCAACTG-3'), c-jun (F 5´-GCATGAGGAACCGCA- TCGCTGCCTCCAAGT-3 and R5´GCGACCAAGTCCTTCCCACTCGTGCA- CACT-3'), NKX3.1 (F 5´- GTACCTGTCGGCCCCTGAACG-3´ and R5´-GCT- GTTA TACACGGAGACCAGG-3´), c-Myc (F 5´-GGCGGGCACTTTGCACTGGA-3´and R5´-TCGCGGGAGGCTGCTGGTTT-3´), KLK2 ( F 5´-AGATGAAGACTCC-AGCCAT-3´ and R 5´-GATACCTTGAAGCACACCA -3´), β-actin (F 5´-CGTGG- GGCGCCCCAG -3´ and R 5´-TTGGCCTTGGGGTTCAGGGGG -3´).

The mRNA amount was determined by using the comparative C_T_ method. Samples were analyzed in triplicates and the data compared with the expression of mRNA in non-treated control which was set as a reference value. 

### Animals and tumor inoculation

Four to six weeks old NMRI male Nude mice from Taconic Europe (Lille Skensved, Denmark) were used in the experiment. The xenograft model was carried out according to the protocol specifically approved by the Ethics committee of Lund university (approval number M 239-10). Mice were kept according to the guidelines given by the Malmö-Lund Ethical Committee. LNCaP cells (2x10^6^ cells) were injected subcutaneously into both flanks resulting in two tumors per mouse. After tumors have developed surgical castration was performed. The animals were assigned into 6 groups: mice were treated with vehicle, Doc, Pac or bicalutamide as a single agent and Doc or Pac combined with bicalutamide. Drugs were administered intraperitoneally (i.p.) (à 20 mg/kg) in 100 µl volume once a week for 5 weeks except for bicalutamide which was administered twice a week. At the time point designated week 0, treatment was initiated and mice were sacrificed one week after the last treatment. Tumors were harvested, fixed in formalin and paraffin embedded for immunohistochemical analysis.

### Immunohistochemistry

Dissected xenograft tumors were fixed in 4% paraformaldehyde and thereafter embedded in paraffin. Four µm thick sections were deparaffinized, rehydrated and microwave treated for 10 min in high pH target retrieval solution (Dako, Glostrup, Denmark) before being processed in automatic Techmate 500 immunohistochemistry staining machine (Dako) using antibodies against AR, c-jun, p-cjun, and Ki-67 (Dako). DAB (3,3'-diaminobenzidine) was used as a chromogenic substrate and slides were counterstained with hematoxylin. The specimens were viewed with an Olympus AX 70 microscope at a magnification of x 10. An arbitrary semiquantitative scoring was applied to evaluate the staining signals and scored in three categories; negative staining (0), weak but detectable staining of some or all cells (1), moderate staining (2) or strong staining (3). At least two sections per tumor were determined and the scores were representative for at least 70% of the area of tissue analyzed.  

### Sub-cellular fractionation

Cellular fractionation was performed according to the protocol supplied with the NE-PER nuclear and cytoplasmic extraction reagents (Pierce, Rockford, IL). LNCaP cells were exposed to Doc or Pac for 6, 24 and 48 hours or remained untreated. Fractions were boiled with SDS sample buffer, subjected to SDS-PAGE, and transferred to a nitrocellulose membrane and probed as indicated with primary antibodies to AR, β-actin or lamin A, all from Santa Cruz, followed by secondary antibodies (IRDye 680,  IRdye 800) which were obtained from Li-Cor Biotechnology (Nebraska, USA). Proteins were detected by Odyssey® Infrared Imaging System.

### Statistical Analysis

Results were obtained from at least three experiments performed and are expressed as the mean ± SD. Statistical significance was determined with unpaired Student's t-test. Values of p < 0.05 were considered significant. 

## Results

### c-jun overexpression induced resistance to Doc treatment

**Figure 1:**
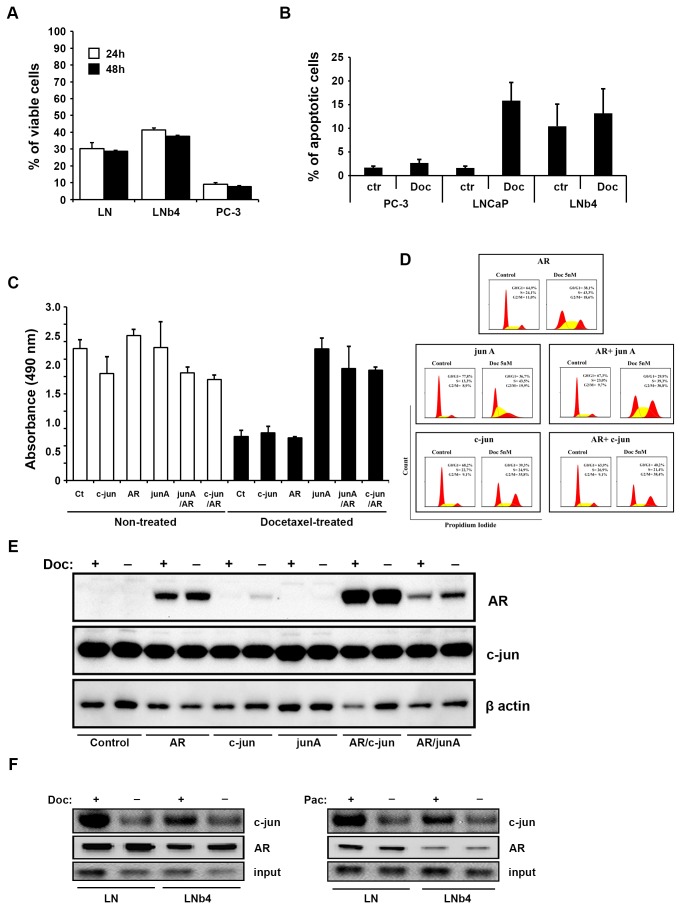
Cell proliferation, apoptosis, cell cycle analysis and protein analysis. (A) Cell viability examined by trypan blue exclusion in LNCaP (LN), LNb4 and PC-3 cells. (B) Determination of apoptosis by flow cytometry. After treatment of cells with Doc, the extent of apoptosis was assessed by annexin V/7AAD assay. (C) MTS assay in PC-3 cells transfected as indicated in figure and treated 48 hours with 5 nM Doc. (D) Graphs from flow cytometry in cells mentioned in (C) demonstrating cycling cell population stained with propidium iodide. Cells were treated with Doc for 48 h or remained untreated and sorted into different stages of mitosis by flow cytometry. (E) Western blotting analysis to show protein expression in transfected cells mentioned in (C). (F) Analysis of protein-protein interaction determined by immunoprecipitation in the presence or absence of Doc or Pac in LN and LNb4 cells. Cells were immunoprecipitated with anti-AR (441) antibody and membranes were probed with anti-c-jun antibody. For control, 20 µg of the cell lysate was used as input and equal loading was confimed with AR antibody.

The function of c-jun as a transcription factor related to proliferation has been reported in prostate cancer before [22]. To investigate the impact of c-jun on Doc treated prostate cancer cells, experiments were carried out in LNCaP, LNb4 and PC-3 cells. We observed that LNCaP cells treated with Doc were more resistant to the treatment at indicated time points compared to PC-3 cells (30 % vs 9%, p=0.003) (Figure 1A). Although we did not detect a statistically significant difference between LNCaP and LNb4 we could see a tendency that long term treatment with bicalutamide increases resistance (Figure 1A). Furthermore apoptosis analysis was performed by using Annexin V/7AAD confirming less apoptosis in LNb4 cells exposed to Doc compared to parental LNCaP cells 

(Figure 1B). To examine whether c-jun is involved in the response to Doc therapy, PC-3 cells were transfected with c-jun, c-jun mutant (junA) and co-transfected with AR (AR/cjun, AR/junA, respectively) plasmids and MTS assay carried out. PC-3 cells transfected with junA showed a statistically significant increase (p=0.01, junA vs control) in cell proliferation (Figure 1C) whereas transfection with AR decreased cell viability comparable to Doc treated control cells. Although there was no statistically significant difference between Doc treated control cells and c-jun transfected PC-3 cells there was a clear tendency to increased viability in the latter. Interestingly, co-tranfection of AR in PC-3 cells overexpressing of c-jun or junA did not abrogate this effect on proliferation (Figure 1C) suggesting a role of c-jun as a potent antiapoptotic factor. Cell cycle analysis was then carried out and displayed cell arrest in G2-M phase in Doc treated PC-3 cells (Figure 1D). We observed that PC-3 cells transfected with AR or junA showed a similar trend with a marked decrease of percentage of cells in G2-M phase (18,6% and 19,9%, respectively), while S phase was increased (43,4% and 43,5%, respectively), suggesting that the phosphorylation of c-jun plays a crucial role in cell response to Doc (Figure 1D). 

We next examined the protein expression in the transfected PC-3 cells. Western blotting analysis showed a marked increase in AR protein in cells co-transfected with AR/cjun compared to cells transfected with AR alone or AR/junA (Figure 1E). These results suggest that the interaction between AR and c-jun is dependent on the available phophorylation site 63/73. To further evaluate the effect of taxane agents on endogenous c-jun and AR immunoprecipitation was performed in LNCaP and LNb4. We found an interaction between AR and c-jun in both cell types, which was enhanced by Doc and Pac treatment (Figure 1F). These findings confirm that there is a physical interaction between c-jun and AR which regulates the effect of taxane therapy in PC cells. 

### Effects of c-jun siRNA on Doc response

**Figure 2:**
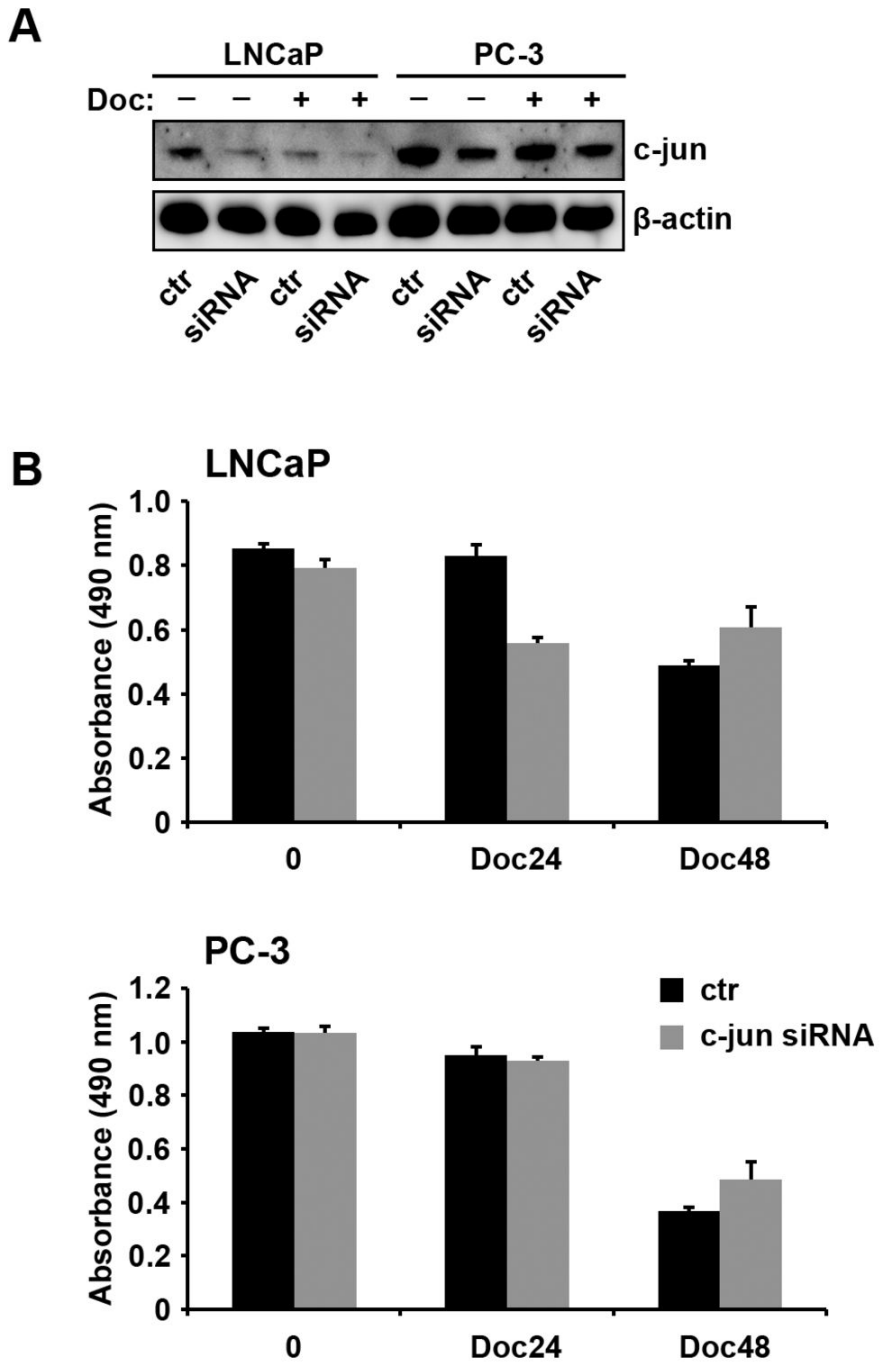
Determination of c-jun siRNA. (A) Western blotting analysis showing transfection efficiency of siRNA c-jun in both PC-3 and LNCaP cells. (B) Cell proliferation in LNCaP (upper panel) and PC-3 (lower panel) cells transfected with c-jun siRNA or nontargeted (control) vector. Cells were treated with Doc for 24 and 48 hours. LNCaP cells harbor c-jun siRNA showed a significant decrease in proliferation (p=0.002) while PC-3 cells were not affected.

 To investigate whether c-jun downregulation can alter PC cell response to Doc we transfected PC-3 and LNCaP cells with siRNA or nontargeted siRNA, which served as a control (Figure 2A). Cells were exposed to Doc for 24 or 48 hours and subjected to MTS assay. c-jun was significantly downregulated although not completely diminished in both cell lines (Figure 2A). LNCaP cells silenced with siRNA showed a clear decrease in viability after 24 hours to Doc exposure compared to parental LNCaP cells (p=0.002). In PC-3 cells we did not observe a significant change in cell viability at the same time point. After 48 hours the effect of c-jun silencing decreased and cells recovered as shown in Figure 2B.

### Taxane-induced p-cjun expression is independent on JNK pathway in PC cells

**Figure 3:**
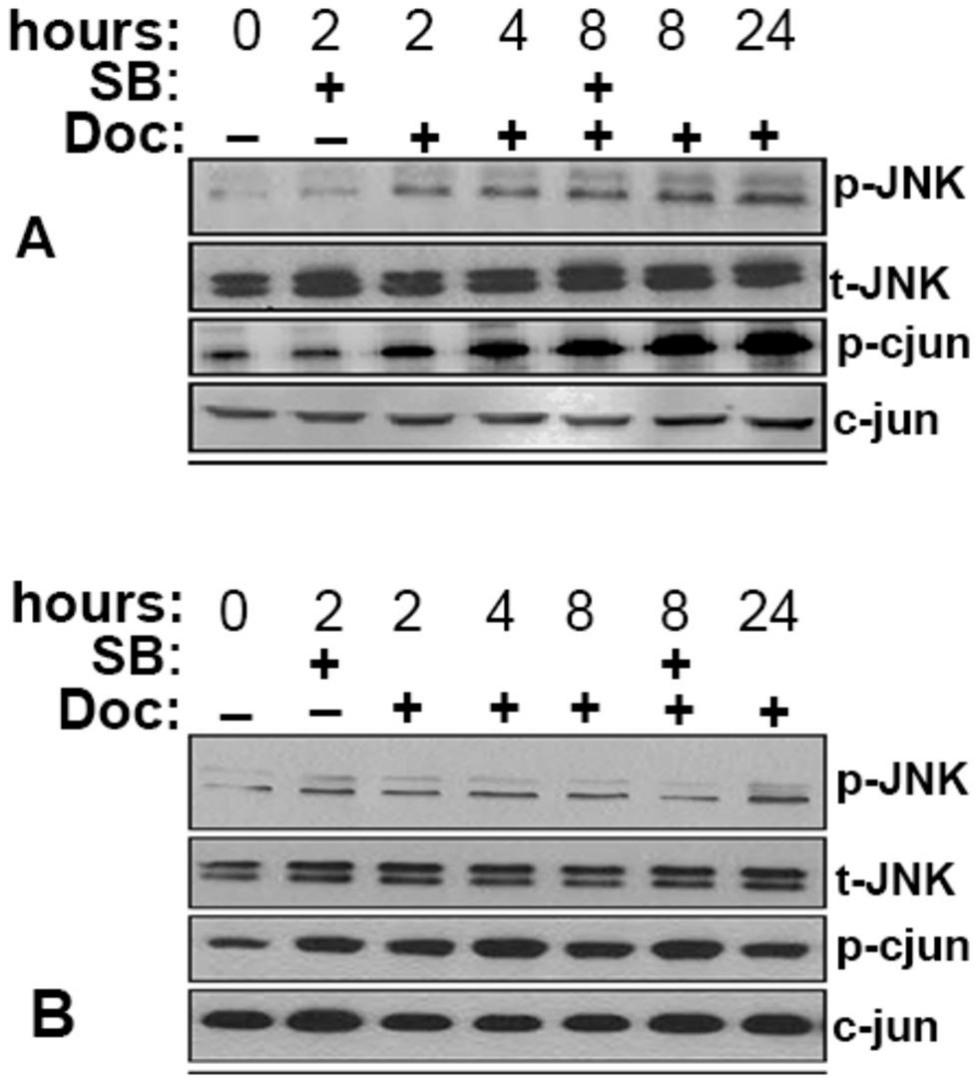
Taxane-induced p-cjun expression is independent on JNK. Western blotting analysis of (A) PC-3 and (B) LNCaP cells exposed to Doc (5 nM) for up to 24 hours or remained untreated (0). PC-3 and LNCap cells were seeded in 24-well plates at a density of 50,000 cells per well and treated with Doc and Pac or in combination with 500 nM of JNK inhibitor (SB) as indicated.

 It has been reported that Doc chemotherapy induces its effect through selectively activating JNK in cancer cells [23]. Once activated, JNK phosphorylates and activates a number of transcription factors such as c-jun. To address this question we analyzed whether JNK signaling pathway has a role in Doc-induced prostate cancer cell death. PC-3 and LNCaP cells were treated with 500 nM of JNK inhibitor for 2 or 8 hours, whereas Doc and Pac treated cells performed in time points 2, 4, 8 and 24 hours. As shown in Figure 3A c-jun phosphorylation was induced 2 hours after exposure to Doc in PC-3 cells, while JNK phosphorylation appeared 24 hours after exposure in PC-3 cells only (Figure 3A). In LNCaP cells exposed to Doc no marked changes in JNK phophorylation was observed at any time point of drug treatment (Figure 3B). This suggests that the JNK activation may be a secondary effect of Doc treatment and c-jun phosphorylation is a primary target of Doc. Especially in AR expressing LNCaP cells, a direct interaction with AR that cause rapid induction of c-jun phosphorylation cannot be excluded. Furthermore, similar results were obtained in the aforementioned cells exposed to Pac (figure not shown). It is worth mentioning that a basal level of p-cjun in non-treated cells was expressed in both cell lines.

### Taxane agents alter protein levels of AR and PSA

**Figure 4:**
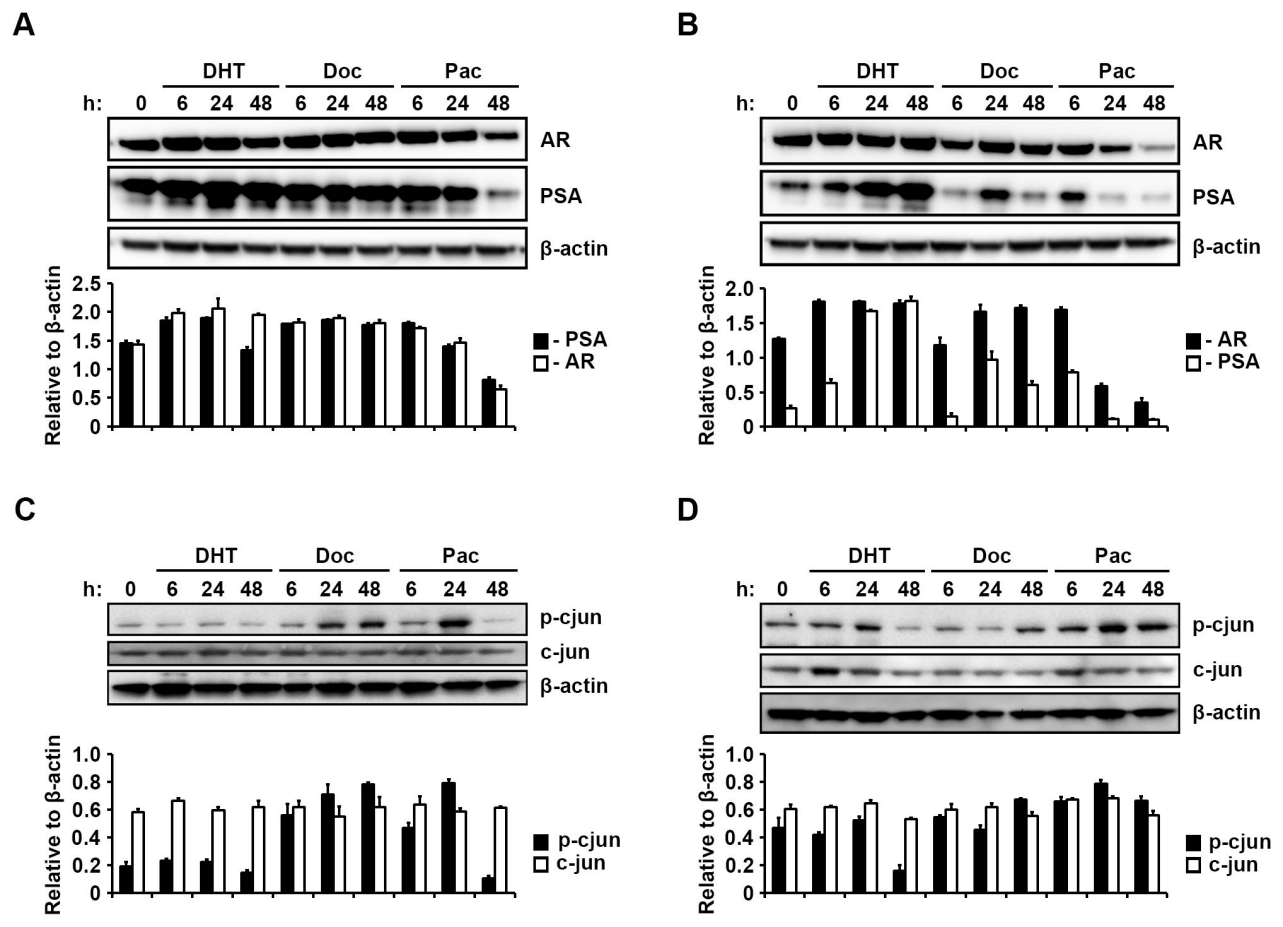
Taxane altered AR, PSA and p-cjun protein level. Western blotting analysis of the expression of AR and PSA in (A) LNCaP and (B) LNb4 cells. Cells exposed to 5 nM of Doc, 5 nM of Pac or 10 nM of DHT for up to 48 hours or remained untreated. The same lysates were used to analyse the expression of p-cjun and c-jun in (C) LNCaP and (D) LNb4 cells. Protein expression levels were evaluated by densitometric analysis (lower panels).

Further experiments were exclusively carried out in LNCaP and LNb4 cells only. Based on our above result we further examined how taxane therapy affects the expression of AR regulated genes such as PSA on protein level. We observed a concomitant increase of AR and PSA protein levels in Doc treated LNCaP cells (p=0.01, Doc 24 vs control) (Figure 4A). However, in Pac treated LNCaP cells a gradual decrease of AR protein was seen concomitantly with PSA level after 48 hours (Figure 4A). Interestingly, the increase of AR protein level in LNb4 cells was more pronounced by Doc treatment, whereas PSA protein level was clearly decreased after 48 hours (p=0.02, Doc 24 vs Doc 48). Exposure of LNb4 cells to Pac led to a markedly decrease in AR and PSA protein expression (Figure 4B). Expression of p-cjun differed in LNCaP and LNb4 (Figure 4C,D). In LNCaP cells Doc treatment increased p-cjun expression gradually (p=0.04, Doc 6 vs Doc 48), whereas in Pac treated cells an increase at 24 hours was seen, which was followed by a marked decrease at 48 hours (Figure 4C). However, in LNb4 cells Pac treatment resulted in a time-dependent induction of p-cjun, whereas Doc treatment failed to do so (Figure 4D). No marked changes in c-jun levels were observed in both cell lines and under all conditions examined. 

### Effect of taxane treatment on c-jun and AR mRNA expression

**Figure 5:**
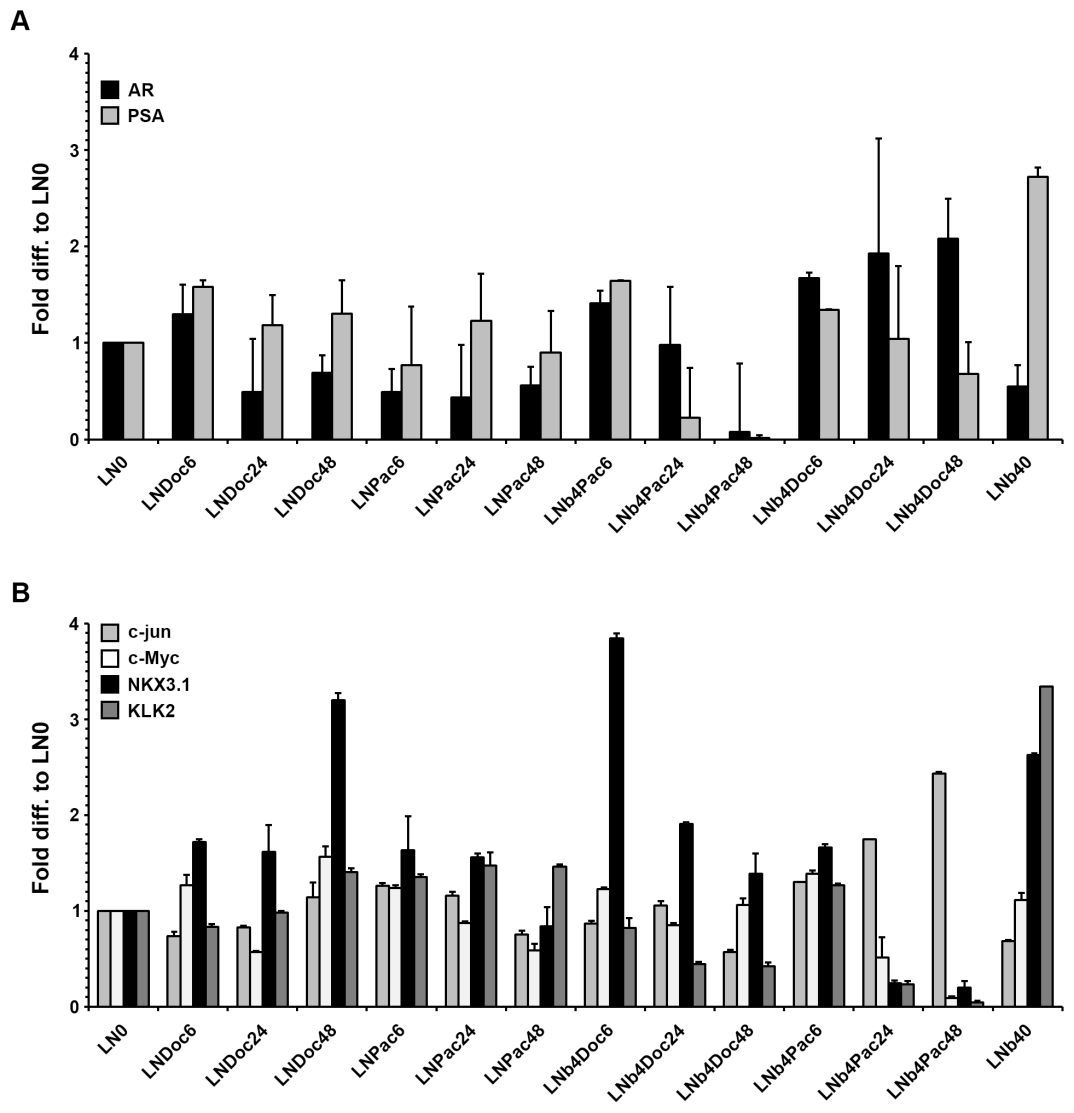
Quantitative real-time RT-PCR. Expression of (A) AR and PSA and (B) c-jun, KLK2, NKX3.1 and c-Myc mRNA levels in response to Doc or Pac was evaluated by quantitative real time PCR (qPCR). The relative mean mRNA expression level of AR regulated genes was measured by quantitative real-time RT-PCR in triplicates.

To investigate the RNA changes due to taxane treatment, QRT-PCR was carried out in LNCaP and LNb4 cells. Cells were treated as indicated in Figure 5. Although we observed an initial induction of AR- and PSA mRNA in Doc treated LNCaP cells, this did not occur in Pac treatment. Interestingly, LNb4 cells showed a continuous increase of AR mRNA expression in a time dependent manner when treated with Doc, whereas PSA mRNA was negatively correlated to AR mRNA expression (Figure 5A). There was a statistically significant difference in the AR and PSA mRNA level at 24 and 48 hours of exposure to Doc (p=0.05 and p=0.003, respectively). However, in Pac treated LNb4 cells we observed an initial induction of AR and PSA mRNA which decreased to a significant level after 48 hours. In contrast, c-jun mRNA expression increased significantly in Pac treated LNb4 cells (p=0.03), which could not be shown in Doc treated cells (Figure 5B). As expected KLK2 mRNA expression showed a similar pattern like PSA mRNA in all cells treated. Further analyzes on c-myc and NKX3.1 was performed as shown in Figure 5B. The results show no significant trend in c-Myc mRNA expression regardless of taxane used and time of treatment. Nevertheless we observed an increased expression of NKX3.1 mRNA in all Doc treated cells, while this was less pronounced in Pac treated cells (Figure 5B).

To summarize our results show a clear correlation between AR, c-jun and PSA in taxane treated cells. The outcome of the treatment is dependent on the status of AR receptor and c-jun mRNA expression and the taxane used. 

### Animal model

**Figure 6:**
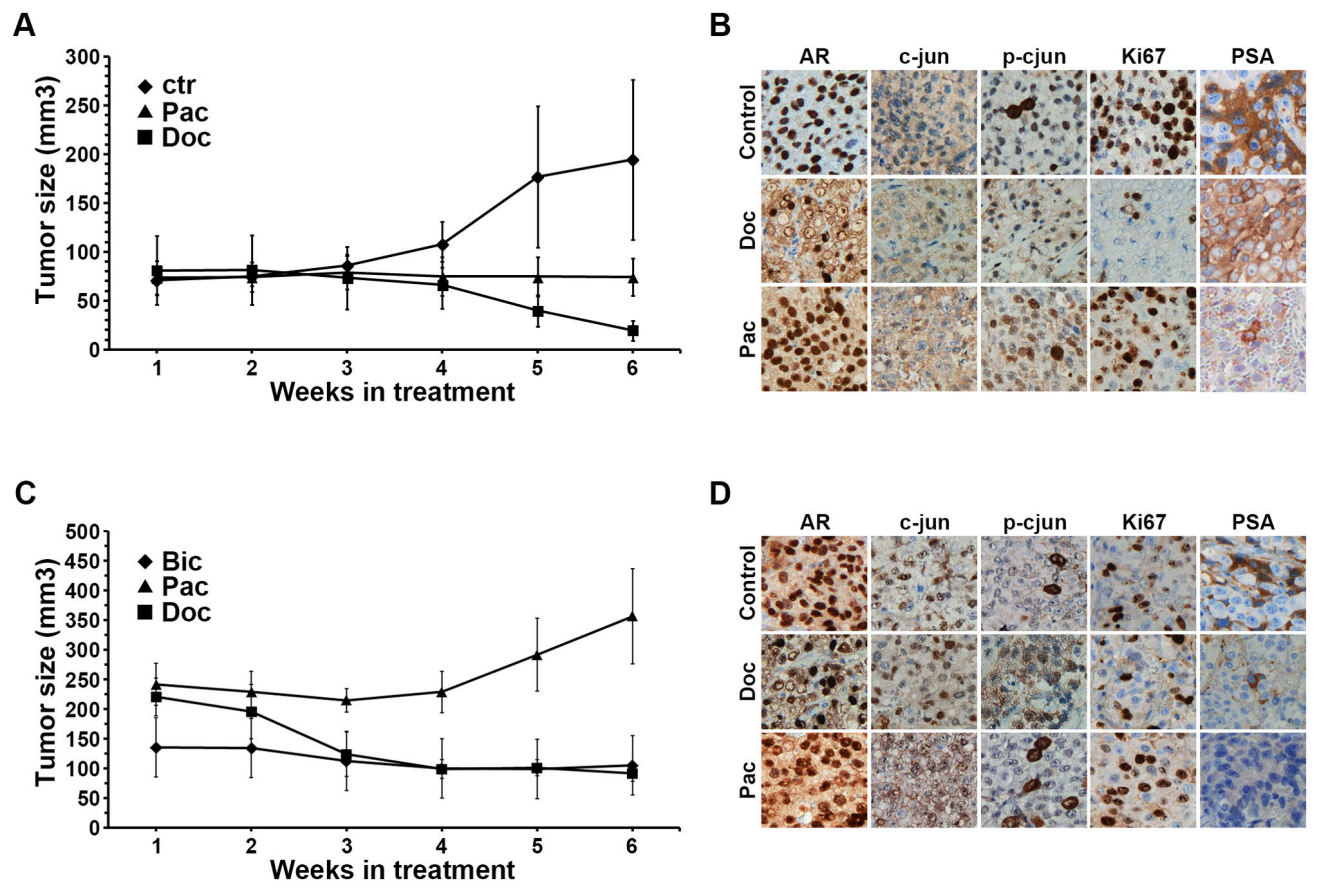
Tumor growth graphs and immunohistochemical analysis of dissected tumors from NMRI-nude mice bearing implanted LNCaP cells. (A) Growth curve of mice treated with Doc or Pac alone, Doc vs ctr (p=0.04). (B) Corresponding immunohistochemical staining of tumor tissue. (C) Growth curve of mice with combined treatment, Doc/bicalutamide (Bic) vs Pac/Bic (p=0.003). (D) Corresponding immunohistochemical staining of tumor tissue. Note that p-cjun was differentially expressed in mice treated with Doc and Pac. Ki67 expression was higher in Pac compared to Doc treated mice.

To assess the therapeutic response of the prostate cancer cell line LNCaP in vivo we established an animal model as indicated in Figure 5. Mice were treated with Doc or Pac as single agent or in combination with bicalutamide. A statisitically significant reduction in tumor size of mice treated with Doc alone was shown (p=0.04, Doc vs ctr) (Figure 6A). In mice treated with Pac a stabilization but no decrease in tumor volume was observed (Figure 6A). There was no significant difference observed between Doc treated mice and mice receiving combined treatment with bicalutamide (Figure 6C). Interestingly, tumors in mice treated with Pac and bicalutamide tumors started to regrow after an initial stabilization (Figure 6C). There was a statistically significant tumor size reduction in Doc/bic compared to Pac/bic treated mice (p=0.003).

 We next examined the expression of c-jun, p-cjun, AR and Ki67 proteins in the tumor tissues harvested to assess the effect of drug treatment. There was a significant change in nuclear AR levels in animals treated with Doc alone or Doc/bic (Figure 6B and 6D, respectively). In both treatment groups approximately 60% of the cell population displayed a cytoplasmic translocation of AR, compared to mice in control group (Figure 6B). In contrast, mice treated with Pac or combination therapy the cytoplasmic translocation of AR was less pronounced. However, the expression of p-cjun in tissues from control animals displayed an intense staining, whereas we observed that nuclear p-cjun was more distinct in Pac treated mice compared to Doc treated animals where also a cytoplasmic expression was found (Figure 6B). No significant change in the pattern of c-jun expression was seen in control and treated animals. Ki67 expression was more pronounced in Pac treated mice which reflects the poor response of tumors to this taxane in vivo.

To confirm the cytoplasmic translocation of AR by taxane therapy in vitro, Western blotting experiments were performed in LNCaP cells (Figure S1). In agreement with the in vivo data we demonstrated that independent on the taxane used AR was translocated to the cytoplasm. The translocation was more pronounced in Doc treated cells (upper panel) compared to Pac treated cells (lower panel), especially after 48 hours of treatment.

## Discussion

In this study, we elucidated the role of AR and its coactivator c-jun in the PC cell response to taxane therapy. We have shown that c-jun overexpression in PC-3 cells confers a statistically significant higher resistance to Doc treatment compared to cells co-transfected with AR/c-jun or AR alone. LNCaP cells were more resistant to Doc in the presence or absence of bicalutamide compared to PC-3 cells. Transfection with mutant junA increased viability, suggesting that c-jun and the phophorylation site 63/73 is a key regulator of cell response to taxanes in PC. We have shown for the first time that Doc and Pac treatment differently affects protein and mRNA levels of AR and PSA in parental LNCaP and LNb4 cells. These differences were reflected in tumor growth in the mouse model. Results presented here provide evidence that the effect of taxane treatment is strongly dependent on the c-jun and AR status of the cell line used and on the drug used for treatment. 

It is known that AP-1 transcription factor is multifunctional and sometimes controversially discussed in literature [24]. c-jun has been demonstrated to transduce a mitogenic response and to promote cell growth as a single gene or in cooperation with an activated ras gene [25,26]. Accordingly, we have found that c-jun overexpression (mutant junA) confers resistance to PC-3 cells to Doc therapy. Interestingly, junA was found to be more potent in preventing cell death compared with AR suggesting that the phophorylation site 63/73 is crucial for the interaction and stabilization of AR and c-jun complexes. This finding is in line with previously reported studies indicating that c-jun may act as an antiapoptotic factor rather than proapoptotic in context with exposure to chemotherapeutic agents [14,27]. This was also confirmed in our study by silencing of c-jun in LNCaP cells which resulted in a better cell response to Doc exposure after 24 hour reflected by a decrease in cell viability. However, silencing in PC-3 cells did not significantly inhibit cell growth upon Doc therapy in this study which was also observed by other groups. Choong et al. showed in their study a downregulation on protein level for c-jun in PC-3 cells which did not result in cell growth inhibition [28]. However, they could show that the oligonucleotide Dz13, a DNA enzyme cleaving c-jun mRNA, could effectively downregulate the target gene resulting in a significant growth inhibiton. Interaction of AR and c-jun has been shown by us and others [22,29], and this may provide basis for the reduced sensitivity to Doc treatment exhibited in LNCaP but also in LNb4 cells. Although other authors have reported that the phosphorylation of c-jun by taxane therapy in other cancer cells was induced by JNK pathway, we could not observe this in this study. We found c-jun phosphorylation to be an early event before JNK pathway is activated and we have to speculate that c-jun activation occurs through a yet undefined pathway independent on JNK or is autophosphorylated. 

The effects of taxane therapy on AR obtained in this study was to some extent in line with previous reports [6,9,11,30], however, their studies differ in terms of concentrations of taxanes or in cell lines used for in vitro and in vivo experiments. We have used PTEN negative LNCaP and LNb4 cells to investigate the impact of Doc and Pac in a head to head study in vitro and in vivo. The inverse correlation of AR and PSA observed in Doc treated LNb4 cells was an important finding in this study as response to chemotherapy in patients treated by Doc therapy still relies on PSA measurements. This study confirms also the results published by Kuroda et al., where PSA as a suitable surrogate marker for response to Doc treatment was critically discussed [31]. An explanation for this finding might be that AR and c-jun protein interact and can repress the androgenic induction of PSA gene as reported by Sato and coworkers in parental LNCaP cells. They suggested that this repression is regulated by the ratio of AR and c-jun [32]. The fact that we could observe a clear difference between Pac and Doc treated LNb4 cells underlines our hypothesis that the ratio of c-jun and AR is affected differently by this agents and so is also the phosphorylation status of c-jun.

Nevertheless the immunohistochemistry of tumors harvested failed to show a major phosphorylation of c-jun which we observed in our in vitro studies, although p-cjun expression pattern differed in Pac treated tumors in being more distinct localized to the nucleus. The same was also true for the AR pattern as seen in Figure 6. However, the growth curves in Pac/bic and Doc/bic treated mice differed markedly which supports our hypothesis that c-jun acts as a potent antiapoptotic factor even in our in vivo model. The xenograft model shows also clearly that the combined treatment of taxane and bicalutamide does not improve the outcome of therapy rendering the proposed model of AR entrapment by taxane therapy incomplete [6].

 Further work is warranted to investigate all phophorylation sites of c-jun and their interaction with AR to further elucidate taxane specific effects observed in this study as suggested by Weiss et al who has shown that c-jun is under control of repressors which are released upon phosphorylation [15]. 

Taken together c-jun and AR interaction is enhanced by taxane therapy. Androgen receptor was translocated by both taxanes to the cytoplasm (se Figure 6D). The phophorylation of c-jun is not only linked to JNK pathway and occurs in the early phase of taxane treatment by yet undefined mechanism. In castration resistant prostate cancer cells Doc therapy upregulates AR whereas Pac decreases AR. However, PSA expression was decreased in both taxanes, indicating that c-jun was able to repress PSA promoter independent on the taxane used in hormone refractory prostate cancer cells. Given the fact that taxane responders in clinical practice are yet defined by PSA decrease (<50%) one should consider that this might not reflect the true AR receptor activation in the patient.

## Supporting Information

Figure S1 **Western blotting analysis showing AR translocation to cytoplasm in LNCaP cells treated with Doc (upper panel) and Pac (lower panel).**
(TIF)
