# Correction: Interaction between c-jun and Androgen Receptor Determines the Outcome of Taxane Therapy in Castration Resistant Prostate Cancer

**DOI:** 10.1371/journal.pone.0209544

**Published:** 2018-12-18

**Authors:** Martina Tinzl, Binshen Chen, Shao-Yong Chen, Julius Semenas, Per-Anders Abrahamsson, Nishtman Dizeyi

There is an error in the Materials and Methods section under the sub-heading, “Real-time Quantitative PCR Analysis”. The AR primers used in the study appear incorrectly. The correct primers are: F-CCTGGCTTCCGCAACTTACAC and R-GGACTTGTGCATGCGGTACTCA.
