# Supplementary figures and images for: Interaction between c-jun and Androgen Receptor Determines the Outcome of Taxane Therapy in Castration Resistant Prostate Cancer
Source: PLoS One. 2013 Nov 8;8(11):e79573. doi: 10.1371/journal.pone.0079573 (PMC3832643; doi:10.1371/journal.pone.0079573)

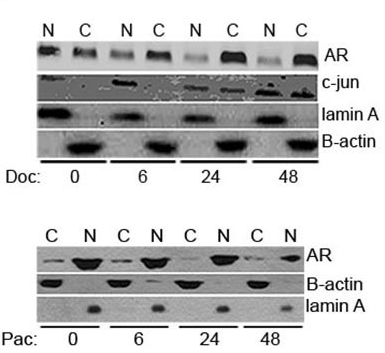

Supplement: Figure S1 — Western blotting analysis showing AR translocation to cytoplasm in LNCaP cells treated with Doc (upper panel) and Pac (lower panel). (TIF) [file pone.0079573.s001.tif]
